# Association between plasma glucuronic acid levels and clinical features in schizophrenia

**DOI:** 10.1192/bjo.2025.20

**Published:** 2025-03-31

**Authors:** Kazuya Toriumi, Mitsuhiro Miyashita, Syudo Yamasaki, Kazuhiro Suzuki, Koichi Tabata, Satoshi Yamaguchi, Satoshi Usami, Masanari Itokawa, Atsushi Nishida, Hidenori Kamiguchi, Makoto Arai

**Affiliations:** Schizophrenia Research Project, Department of Psychiatry and Behavioral Sciences, Tokyo Metropolitan Institute of Medical Science, Tokyo, Japan; Unit for Mental Health Promotion, Research Center for Social Science & Medicine, Tokyo Metropolitan Institute of Medical Science, Tokyo, Japan; Department of Psychiatry, Tokyo Metropolitan Matsuzawa Hospital, Tokyo, Japan; Department of Community Mental Health, School of Medicine, Shinshu University, Nagano, Japan; Department of Psychiatry and Behavioral Sciences, Institution of Science Tokyo, Tokyo, Japan; Center for Research and Development on Transition from Secondary to Higher Education, The University of Tokyo, Tokyo, Japan; Neuroscience Drug Discovery Unit, Takeda Pharmaceutical Company Ltd, Tokyo, Japan

**Keywords:** Schizophrenia, glucuronic acid, negative symptom score, general psychopathology score, psychotropic drugs

## Abstract

**Background:**

Glucuronic acid (GlcA) is crucial in the glucuronidation pathway, facilitating the metabolism and elimination of various substances and drugs. Recent studies have noted elevated GlcA levels in patients with schizophrenia (SCZ) compared with healthy individuals. However, it remains unclear whether this elevation contributes to SCZ pathophysiology or results from medication effects.

**Aims:**

This study investigated the relationship between peripheral GlcA levels and clinical characteristics in patients with SCZ and assess whether these associations persist independently of psychotropic medication effects to provide insight into the potential role of GlcA in the pathophysiology of SCZ.

**Methods:**

Plasma GlcA levels were analysed in 218 patients with SCZ, examining their association with clinical features. The correlation between GlcA levels and symptom severity, assessed using the Positive and Negative Syndrome Scale (PANSS), was analysed in 35 patients. In addition, multiple regression analysis was conducted to adjust for age and psychotropic medication effects.

**Results:**

Significant correlations were observed between GlcA levels and PANSS scores for negative symptoms, general psychopathology and total scores. After adjustment for age and psychotropic medications, significant correlations between GlcA levels and PANSS scores persisted for negative symptoms (adjusted β [95% CI], 13.926 [2.369, 25.483]) and general psychopathology (adjusted β [95% CI], 19.437 [3.884, 34.990]), while the total score was no longer significant (adjusted β [95% CI], 34.054 [–0.517, 68.626]).

**Conclusions:**

Elevated GlcA levels in patients with SCZ are associated with specific symptom severity irrespective of the medication dose, suggesting a potential role of GlcA in SCZ pathophysiology.

Glucuronic acid (GlcA) is a uronic acid derived from glucose oxidised at its sixth carbon. Synthesised primarily in the liver via the uronic acid pathway,^1,2^ GlcA is a key component of a phase II metabolic process known as glucuronidation, which is essential for the detoxification and elimination of waste products, toxins and drugs from the body. Glucuronidation is a process in which GlcA is transferred from uridine diphosphate (UDP)-GlcA to a substrate, catalysed by various UDP-glucuronosyltransferases (UGTs).^3^ A notable example of a glucuronide is conjugated bilirubin, a metabolite of heme found in hemoglobin.^4^

## Regulation of circulating glucuronic acid levels

Circulating GlcA levels are influenced by a complex interplay among metabolic, microbial and pathological factors. Age is a key determinant, with levels increasing over time and correlating with the health span and mortality risk.^5^ Various disease states, such as diabetes,^6,7^ chronic kidney disease,^8^ cancer^9^ and liver disease,^10^ are also associated with elevated GlcA levels owing to disruptions in normal metabolism. Moreover, the intestinal microbiome significantly affects GlcA levels through bacterial β-glucuronidases, which cleave glucuronide conjugates, releasing GlcA into the bloodstream via enterohepatic recirculation.^11^ In addition, endogenous human β-glucuronidase, active in lysosomes, contributes to GlcA elevation by degrading glycosaminoglycans during ageing and extracellular matrix remodelling.^12,13^ Factors such as gastrointestinal pH,^14^ hepatic function^15^ and dietary intake,^16^ including oral ingestion of GlcA or glucuronide conjugates, further modulate circulating levels. Together, these factors underscore the multifaceted regulation of GlcA in the context of health and disease.

## Glucuronic acid as a precursor of pentosidine in schizophrenia

Recently, we identified GlcA as a novel precursor of pentosidine (PEN), an advanced glycation end product (AGE) that accumulates in a sub-population of patients with schizophrenia (SCZ).^17–19^ Our findings indicate that GlcA levels are associated with SCZ diagnosis. A single standard deviation increase in GlcA level was associated with an approximately twofold increase in the odds of SCZ. Furthermore, we observed a decrease in the activity of aldo-keto reductase (AKR), which is responsible for GlcA degradation in patients with SCZ, with a negative correlation between AKR activity and plasma GlcA levels.^17^ These findings suggest that reduced AKR activity in patients with SCZ leads to elevated plasma GlcA levels, potentially promoting PEN accumulation and downstream pathological effects.

## The role of AGEs and glucuronic acid in schizophrenia pathogenesis

AGEs, including PEN, may contribute to the pathogenesis of SCZ through a multifaceted mechanism that includes functional protein alterations, oxidative stress and inflammatory signalling pathways. AGEs are known to significantly modify protein structure and function,^20^ and such modifications in brain proteins may contribute to the pathogenesis of SCZ. AGEs are also associated with oxidative stress, a critical factor linked to the pathophysiology of SCZ.^21^ Furthermore, AGEs can trigger inflammatory responses through the receptor for AGEs (RAGE),^22,23^ whereas GlcA induces inflammation through interactions with Toll-like receptor 4 (TLR4).^24^ The PEN-RAGE and GlcA-TLR4 signalling pathways may drive the heightened inflammatory responses observed in SCZ, potentially contributing to disease onset.^25–27^ However, the precise role of elevated GlcA levels in the pathophysiology of SCZ remains unclear, and the influence of medications, despite their known effects on drug metabolism, remains to be explored.

## Investigating GlcA levels and clinical characteristics in schizophrenia

In this study, we investigated the association between GlcA levels in the peripheral blood of patients with SCZ and clinical characteristics, including symptom severity, as assessed using the Positive and Negative Syndrome Scale (PANSS). In addition, we conducted a multiple regression analysis to examine the effect of psychotropic dosage on this association.

## Method

### Patients

This cross-sectional study aimed to investigate the relationship between GlcA levels and the clinical features in our study population. The patients included in this study were recruited from the cohort used in our previous metabolomic analysis study.^17^ We specifically targeted individuals for whom the PANSS scores were available. This study did not involve the recruitment of a new patient cohort; instead, we used previously collected data. The demographic characteristics of the 218 patients with SCZ are summarised in Supplementary Table S1 available at https://doi.org/10.1192/bjo.2025.20. Two experienced psychiatrists confirmed the participants’ diagnoses according to DSM-IV diagnostic criteria.^28^ Although a structured interview was not used, the psychiatrists conducted face-to-face evaluations to assess symptom severity using the PANSS. They were randomly selected from among in-patients and out-patients. Exclusion criteria included diabetes mellitus (HbA1c ≥ 6.3), renal dysfunction (creatinine > 1.04 mg/dl for men and > 0.79 mg/dl for women, or estimated glomerular filtration rate (eGFR) < 60.0 ml/min/1.73 m^2^), Behçet’s disease and chronic viral hepatitis type C, as these conditions can elevate plasma PEN or GlcA levels. All the participants provided written informed consent.

### Clinical assessment

At the time of blood collection, we assessed clinical variables through face-to-face interviews and cross-referencing medical records. These variables included in-patient and out-patient statuses, family history, education duration, disease onset, disease duration and total duration of hospital stay. For psychotropic drugs, including antipsychotics, antidepressants, anxiolytics, mood stabilisers, hypnotics and anti-Parkinsonism drugs administered to patients with SCZ, we used molar equivalents to standardise the number of drug molecules across medications to evaluate the overall impact of psychotropic medications on GlcA levels. Specific drugs that might influence blood GlcA levels have not been identified; therefore, our analysis focused on the cumulative effect of medication use rather than on individual drug types or dosages. Disease onset was defined as the emergence of clinical symptoms that met DSM-IV criteria. A family history of psychiatric disease was defined as having a first- or second-degree relative who had undergone a psychiatric intervention. Symptom severity for the 35 interviewed patients was assessed using the PANSS, ensuring no symptomatic exacerbation for at least 1 month before the interview. The scores are summarised in Supplementary Table S2.

### Measurement of GlcA

Plasma GlcA levels were obtained from metabolomic analyses conducted in our previous study.^17^ The analyses were performed by Metabolon, Inc. (Durham, NC, USA) using gas chromatography/mass spectrometry (GC/MS) with electron impact ionisation on a Trace DSQ fast-scanning single-quadrupole mass spectrometer (Thermo Scientific, Waltham, MA, USA). The GlcA levels in the patients are summarised in Supplementary Table S2.

### Statistical analysis

Statistical analyses were performed using GraphPad Prism version 10 for macOS (GraphPad Software, San Diego, CA, USA; https://www.graphpad.com) and R software version 4.3.3 for macOS (R Core Team, Vienna, Austria; https://www.r-project.org). The normality of the distribution of GlcA levels and psychotropic medication doses was assessed using the Kolmogorov–Smirnov test. Because both distributions were non-normal, log transformation was applied before further analysis. For the correlation analysis between GlcA levels and clinical features, we selected only patients with available data for each feature. Individual GlcA values were used for these analyses, and no averaging of GlcA values across the cohort was performed. As shown in Table 1, the number of participants (*N*) varied depending on the availability of clinical information. Correlations between GlcA levels and each clinical characteristic, including PANSS scores, were evaluated using Pearson’s correlation test.

**Table 1:** Association between glucuronic acid and clinical features in patients with schizophrenia

	*N*	Scores^a^	*r* [95% CI]^b^	*P*-value^c^
In-patients/out-patients	218	108/110	0.102 [−0.031, 0.232]	0.132
Family history (+/−)	131	71/60	0.049 [−0.124, 0.219]	0.580
Duration of education (year)	126	12.7 ± 2.57	0.058 [−0.121, 0.228]	0.519
Onset (year)	124	24.9 ± 8.21	−0.005 [−0.181, 0.171]	0.954
Disease duration (year)	124	20.3 ± 12.9	0.192 [0.016, 0.356]	0.033*
Number of admissions to hospital	120	3.71 ± 3.62	−0.020 [−0.199, 0.160]	0.826
Duration of hospital stays (year)	119	5.38 ± 9.07	−0.031 [−0.210, 0.149]	0.734
Psychotropics (log(µmol/day))	128	2.72 ± 0.80	0.172 [−0.001, 0.336]	0.052
PANSS positive symptom score	35	17.7 ± 7.30	0.079 [−0.261, 0.402]	0.653
PANSS negative symptom score	35	19.9 ± 9.68	0.409 [0.087, 0.653]	0.015*
PANSS general psychopathology score	35	35.2 ± 13.1	0.406 [0.084, 0.651]	0.016*
PANSS total score	35	72.8 ± 28.5	0.346 [0.014, 0.609]	0.042*

PANSS, Positive and Negative Syndrome Scale.

a . Classification or mean ± standard deviation is shown.

b . Pearson’s product moment correlation coefficients are shown.

c . Statistical differences were determined using Pearson’s correlation test. * denotes statistical significance at *P* < 0.05.

Subsequently, multiple regression analysis was conducted to examine the relationships between GlcA levels and PANSS scores after adjusting for age and psychotropic medication dose effects. Before regression analysis, the assumptions of the regression model were carefully evaluated. Multicollinearity among the independent variables was assessed using variance inflation factors (VIFs), with all VIF values below 5 indicating no significant multicollinearity issues. Residual plots were examined to confirm the linearity and homoscedasticity, ensuring the appropriateness of the regression analysis.

The regression models included log-transformed GlcA levels as the main predictor variable, with age and psychotropic medication dose as covariates. The adjusted *R* ^2^ values were calculated to evaluate the model fit by measuring the proportion of variance in the PANSS scores explained by these variables while penalising based on the number of predictors in the model.

### Study approval

All procedures contributing to this work complied with the ethical standards of the relevant national and institutional committees on human experimentation and the Declaration of Helsinki of 1975, as revised in 2013. All procedures involving human participants were approved by the ethics committees of all participating institutions (Tokyo Metropolitan Matsuzawa Hospital and Tokyo Metropolitan Institute of Medical Science; approval nos. 20-17, 23-12).

## Results

A demographic summary of the control participants and patients with SCZ is presented in Supplementary Table S1. The mean (±s.d.) age of 218 patients with SCZ (male/female 116/102) was 45.9 ± 11.5 years. Using plasma samples from these participants, we previously reported that plasma PEN levels in patients with SCZ were higher than those in controls (*P* < 0.0001) and that patients also showed enhanced GlcA levels compared with healthy participants (*P* < 0.0001).^17^

Here, we investigated the association between plasma GlcA levels and clinical features of patients with SCZ (Table 1). Among the clinical features, GlcA level was significantly associated with disease duration and PANSS scores for negative symptoms, general psychopathology and total scores, indicating a potential association between GlcA accumulation and SCZ symptoms. A correlation was observed between GlcA levels and the mol-equivalent psychotropic dosage, although this was not statistically significant. Given that GlcA is involved in the metabolism of a wide range of drugs, medication dosage may affect the amount of GlcA and its clinical characteristics. Consequently, following multiple regression analysis, GlcA levels remained significantly correlated with PANSS scores after adjusting for age and psychotropic medication doses (Table 2). These findings suggest that GlcA accumulation is related to the pathophysiology of SCZ.

**Table 2:** Association between glucuronic acid and Positive and Negative Syndrome Scale scores, adjusted by age and psychotropic dosage

	Unadjusted model			Adjusted model^a^		
	Standardised β [95% CI]	*P*-value^b^	Adjusted *R* ^2^	Standardised β [95% CI]	*P*-value^b^	Adjusted *R* ^2^
Positive symptom score	2.027 [−7.050, 11.105]	0.653	−0.024	0.691 [−8.459, 9.841]	0.879	0.042
Negative symptom score	13.936 [2.915, 24.956]	0.015*	0.142	13.926 [2.369, 25.483]	0.020*	0.131
General psychopathology score	18.790 [3.810, 33.771]	0.016*	0.140	19.437 [3.884, 34.990]	0.016*	0.146
Total score	34.753 [1.373, 68.134]	0.042*	0.093	34.054 [−0.517, 68.626]	0.053	0.104

a . Adjusted for age and psychotropic dosage.

b . * denotes statistical significance at *P* < 0.05.

## Discussion

This study investigated the relationship among GlcA levels, disease duration and PANSS symptom scores in patients with SCZ. Our findings suggest a potential association between GlcA levels and SCZ pathophysiology (Table 1). Previously, we reported a significant elevation in plasma GlcA levels in patients with SCZ compared with healthy controls.^17^ A one standard deviation increase in GlcA level approximately doubles the risk of SCZ. This elevation aligns with the previous research findings.^29^ In addition, we showed that GlcA levels were associated with negative symptoms and general psychopathology but not with positive symptoms. Negative symptoms are poorly ameliorated by existing antipsychotic medications and have been implicated in impaired functioning.^30^ GlcA may be involved in the pathophysiology of patients with poor response to medication.

A potential molecular mechanism underlying the onset of SCZ involving GlcA may involve AGEs. Previously, elevated levels of PEN have been demonstrated in the peripheral blood of a subset of patients with SCZ compared with healthy controls.^18,19^ This finding has been corroborated by several independent studies.^31–33^ Moreover, our recent investigations indicated that PEN accumulation in SCZ originates from GlcA.^17^ We observed a positive correlation between plasma GlcA and PEN levels, with higher GlcA concentrations in patients with SCZ and elevated PEN levels. Notably, elevated PEN levels derived from GlcA accumulation are associated with severe clinical manifestations, including higher rates of admission to hospital, prolonged stay and increased use of antipsychotic medications.^34^ These results strongly implicate PEN accumulation as a pathological mechanism in SCZ, which is potentially mediated by GlcA accumulation.

Several antipsychotics, including haloperidol and olanzapine; antidepressants, such as imipramine; and mood stabilisers, such as valproic acid, undergo metabolism and elimination via glucuronidation pathways.^35^ Although the administration of these drugs could theoretically increase blood GlcA levels owing to enzyme induction, our study demonstrated significant correlations between GlcA levels and PANSS scores even after adjusting for psychotropic medications (Table 2). These findings are consistent with those of previous metabolomic studies that observed elevated peripheral blood GlcA levels in unmedicated patients with SCZ compared with healthy individuals, with reductions upon antipsychotic treatment.^29^ This suggests that GlcA accumulation precedes the onset of SCZ, independent of medication use. However, determining whether GlcA accumulation in patients with SCZ is caused by medication or disease severity remains challenging in cross-sectional studies. Although the direct impact of psychotropic drugs on plasma-free GlcA remains uncertain, it is known that non-glucuronidable xenobiotic administration increases GlcA concentration.^1^ Further investigations involving longitudinal assessments of pre-onset adolescents are imperative to elucidate the effect of psychotropic medications on GlcA accumulation in SCZ.

Our multiple regression analyses revealed varying degrees of model fit across different PANSS components. The occurrence of negatively adjusted *R*-squared values, particularly in models involving positive symptoms, indicated that the relationship between GlcA levels and these symptoms is complex and is likely influenced by factors beyond those included in our current model. This poor model fit for positive symptoms aligns with our finding that GlcA levels were not significantly associated with positive symptoms, suggesting that different pathophysiological mechanisms underlie positive symptomatology in SCZ.

In a previous study, red blood samples from this cohort were used to investigate the potential role of AKR in GlcA degradation.^17^ Our findings revealed a significant reduction in erythrocyte AKR activity in patients with SCZ compared with controls. In addition, we observed a negative correlation between AKR activity and plasma GlcA levels. Furthermore, genetic analysis identified a variant of the *AKR1A1* gene that is more prevalent in SCZ and significantly diminishes AKR activity.^36^ Studies on *Akr1a*-knockout mice demonstrated GlcA accumulation in peripheral blood,^37^ whereas AKR1A inhibition in mice increased urinary GlcA excretion.^38^ These findings suggested a link between decreased AKR activity and elevated GlcA levels in patients with SCZ. An alternative explanation for the observed increase in GlcA levels is the action of the microbiome. Certain gut bacteria can cleave GlcA from glucuronidated xenobiotics and biomolecules, facilitating its reabsorption into the bloodstream via enterohepatic circulation.^11^ The composition of the gut microbiota directly influences this process, as different microbial species exhibit varying β-glucuronidase activities.^39^ Indeed, several studies have reported alterations in the gut microbiota in SCZ,^40^ suggesting that changes in microbiota composition contribute to elevated GlcA levels.

This study had some limitations that should be acknowledged to better contextualise the findings. These limitations pertain to the sample size, statistical power and the inability to account for all potential medication effects. Furthermore, this study represents preliminary research, and the findings should be interpreted with caution owing to the exploratory nature of the analyses. First, the limited sample size compromised statistical power, particularly in the PANSS analysis, where interview data were accessible for only 35 patients. This limitation reduces the precision of the estimates and may introduce sampling bias, potentially affecting the generalisability of the findings. According to a recent study,^41^ a minimum sample size of 40 is recommended for robust statistical analyses, further emphasising the need for validation in larger cohorts, necessitating validation in a larger cohort. Second, we were unable to consider the effects of all the medications. If enzyme induction is considered, non-psychotropic medications, including headache and laxative medications, may also increase GlcA levels. However, it was not possible to track all medications taken by the patients. Therefore, a fully dose-controlled analysis of the untreated group is required.

In conclusion, this study revealed significant correlations between plasma GlcA levels in patients with SCZ and disease duration, as well as PANSS scores for negative symptoms, general psychopathology and total scores. Furthermore, GlcA levels still exhibited a significant correlation with PANSS scores after adjusting for psychotropic medication dosage. Future studies should elucidate the precise mechanisms through which GlcA influences SCZ symptoms to pave the way for targeted therapeutic interventions aimed at modulating GlcA levels to ameliorate symptom severity and enhance patient outcomes.

## Supporting information

Toriumi et al. supplementary material Toriumi et al. supplementary material

## Data Availability

All data will be made available in the manuscript and the supplementary tables.

## Supplementary material

The supplementary material for this article can be found at https://doi.org/10.1192/bjo.2025.20
